# Supplementary material for: Carbon-doped SnS2 nanostructure as a high-efficiency solar fuel catalyst under visible light
Source: Nat Commun. 2018 Jan 12;9:169. doi: 10.1038/s41467-017-02547-4 (PMC5766557; doi:10.1038/s41467-017-02547-4)
Supplement: Supplementary file 1 — Supplementary Information [file 41467_2017_2547_MOESM1_ESM.pdf]

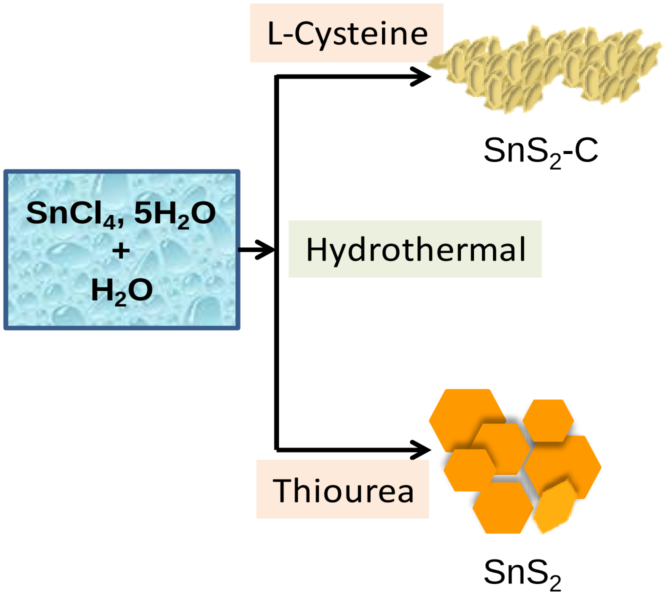

Supplementary Figure 1: Schematic scheme. Hydrothermal synthesis process for SnS_2-C and SnS_2.

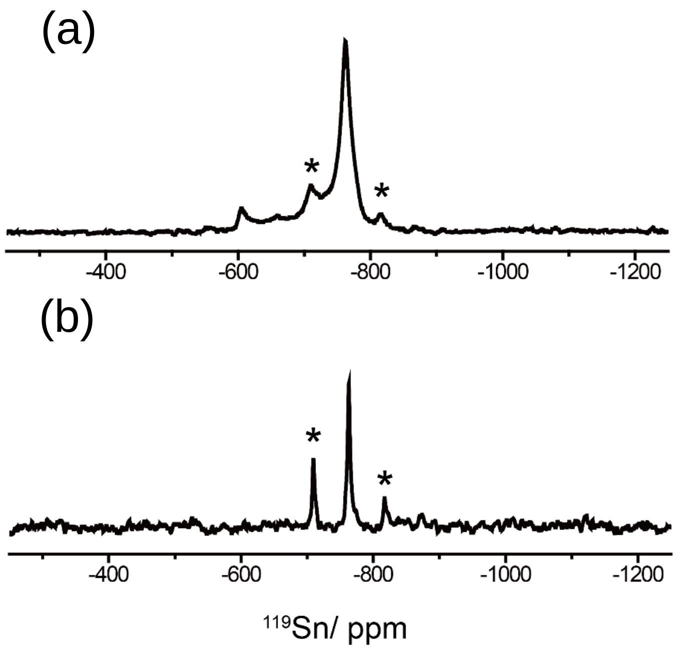

Supplementary Figure 2: ^{119}Sn MAS NMR spectra. (a) SnS_2 -C synthesized by L-cysteine as the source of sulfur and (b) commercial SnS_2 (MKN-SnS_2-900, purchased from M K Implex Corp. Canada), using MAS spinning frequency, 10 kHz; spectral width, 500 kHz; pulse width, 4.5 ms, number of transients, 12,288 for (a) and 3,072 for (b). The asterisks indicate spinning sidebands.

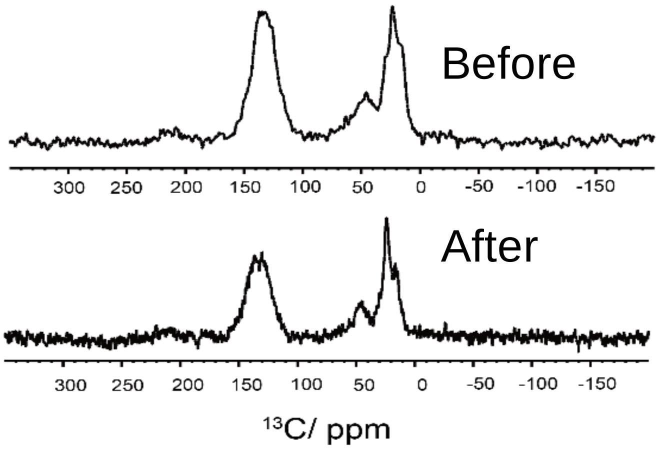

Supplementary Figure 3: ^{13}C NMR analysis. ^{13}C CPMAS NMR spectra of SnS_2-C before and after CO_2 reduction reaction under light. MAS spinning frequency, 10 kHz, was used in these measurements

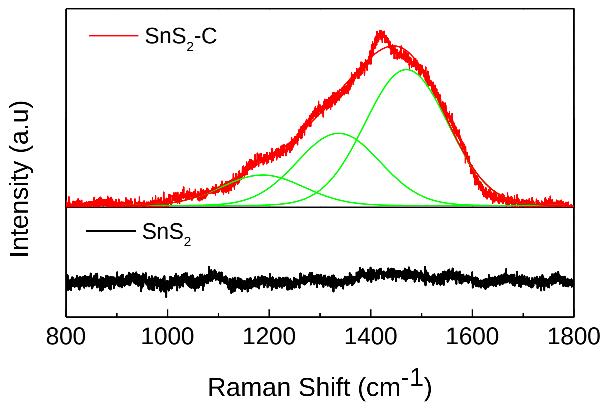

Supplementary Figure 4: Carbonaceous matters analysis in the SnS_2-C. Raman spectra of the SnS_2 -C and SnS_2 (800 to 1800 cm^{-1} Raman shift).

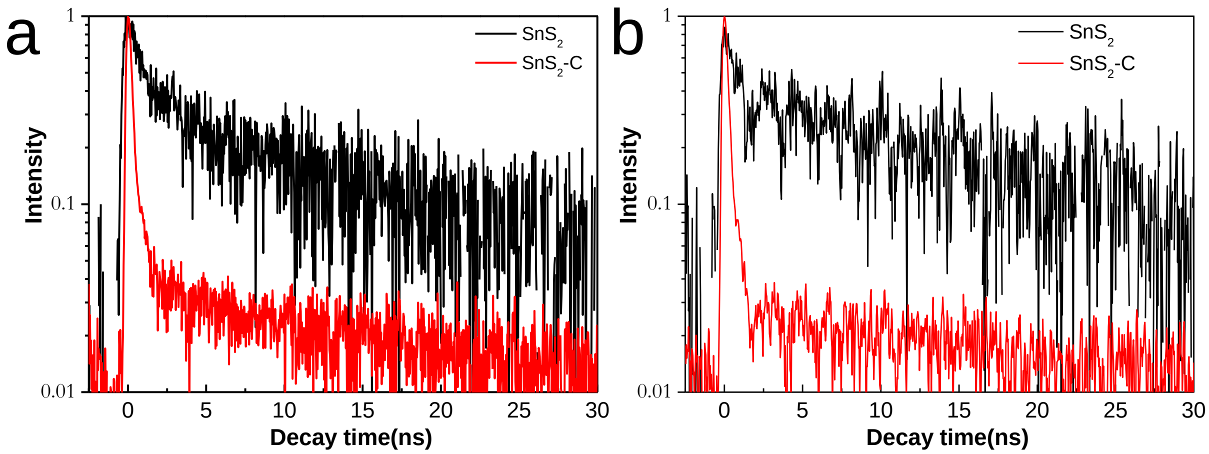

Supplementary Figure 5: Time-resolved photoluminescence (TRPL) measurements. TRPL spectra of SnS_2 and SnS_2-C at (a) 493 and (b) 548 nm emissions respectively.

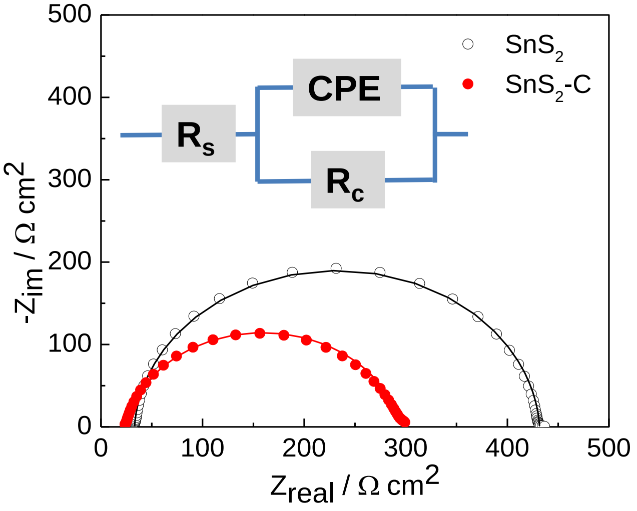

Photocatalyst	R_c (Ω/cm^2)
SnS_2	398.7
SnS_2-C	270.9

Supplementary Figure 6: Impedance analysis. Nyquist plots of SnS_2 and SnS_2-C coated on FTO electrodes at frequencies ranging from 0.01 to 10^5 Hz (1.2 V vs NHE).

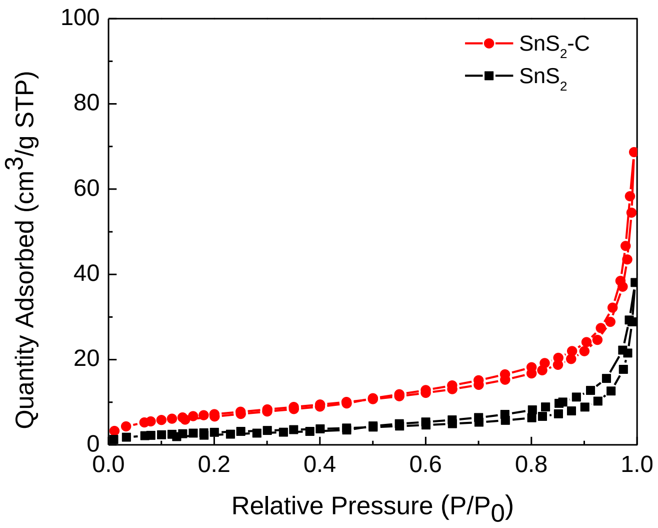

Supplementary Figure 7: Nitrogen adsorption–desorption isotherm measurements. Brunauer–Emmett–Teller (BET) specific surface area of the SnS_2-C and SnS_2.

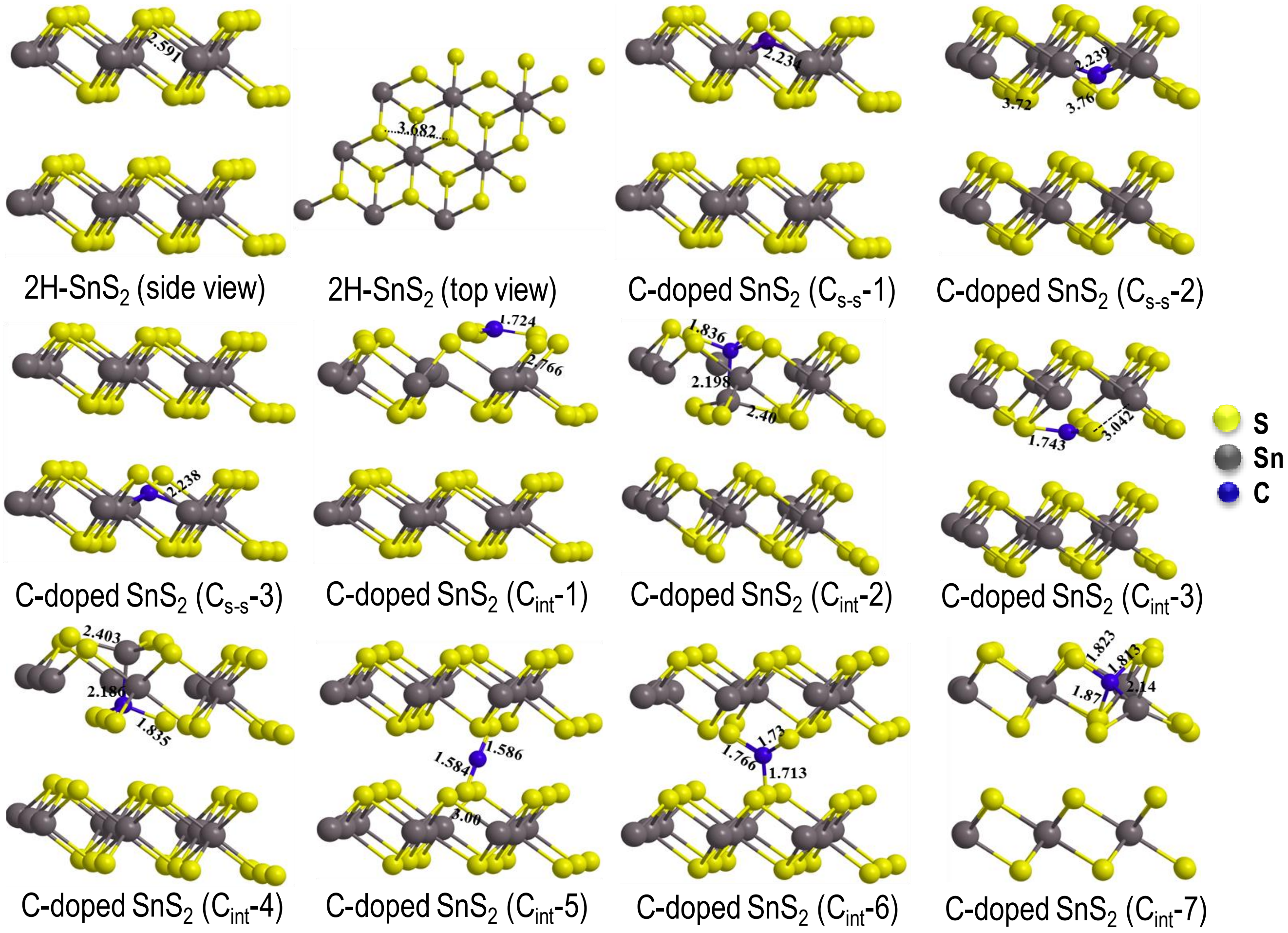

Supplementary Figure 8: Model structures of SnS_2 and C-doped SnS_2. Supercell model and partial geometries from the structurally optimized 2H-SnS_2 and C-doped SnS_2.

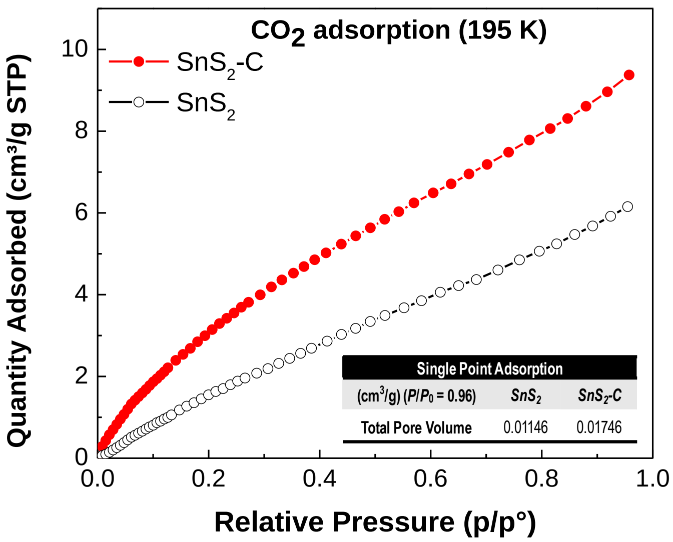

Supplementary Figure 9: CO_2 adsorption isotherm study. CO_2 adsorption measurement at 195 K for SnS_2 and SnS_2-C respectively.

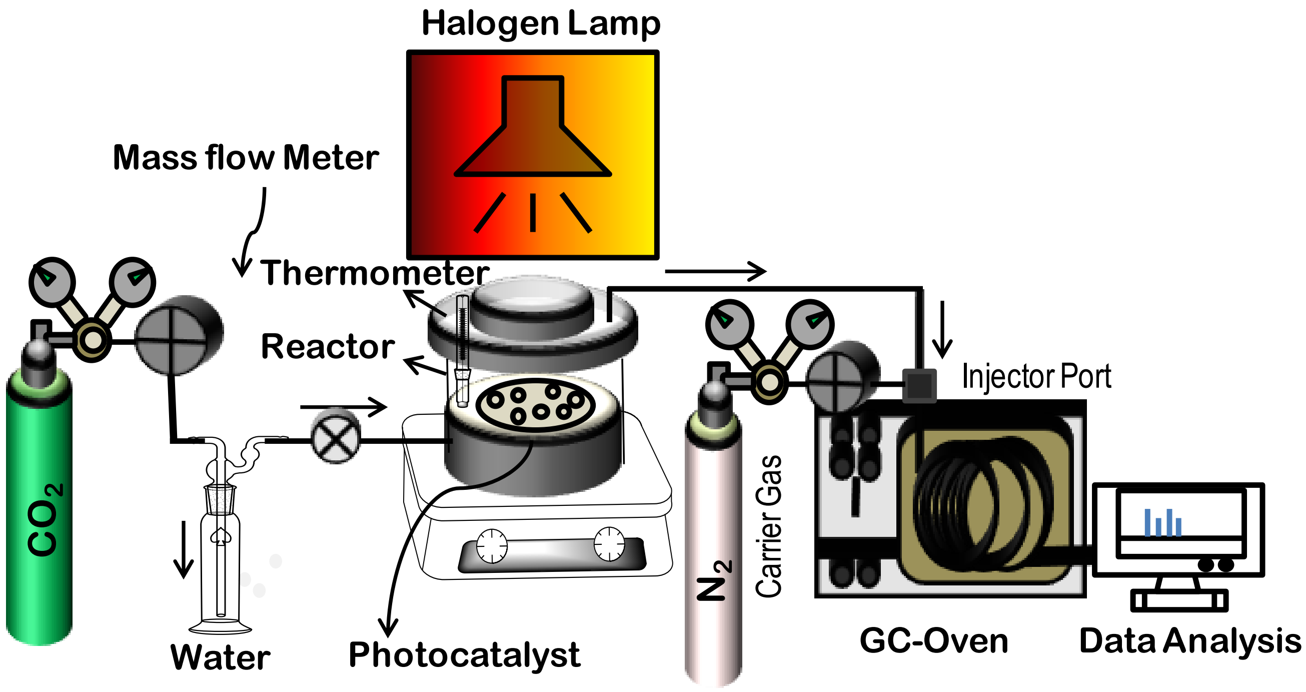

Supplementary Figure 10: Experimental setup. Schematic diagram of the photocatalytic reduction of CO_2 with the photocatalysts obtained by the hydrothermal method.

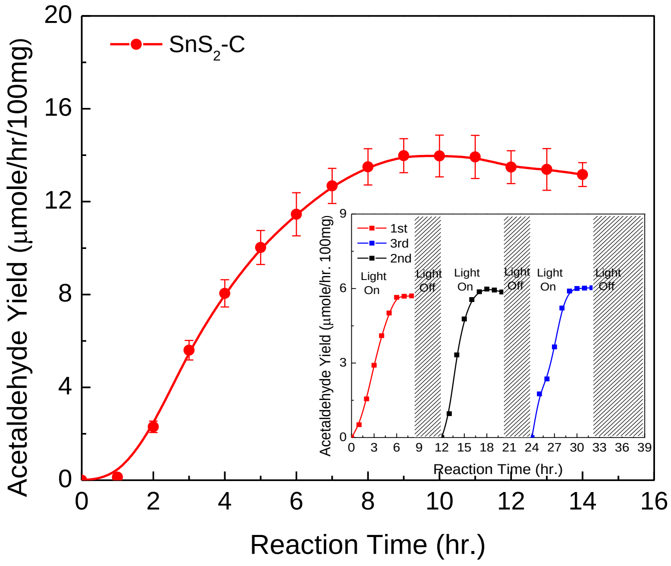

Supplementary Figure 11: Photocatalytic stability study of SnS_2-C photocatalyst. Photocatalytic performance and acetaldehyde yield every hour for SnS_2-C (inset: Stability study via consecutive cycle test using AM 1.5 light source).

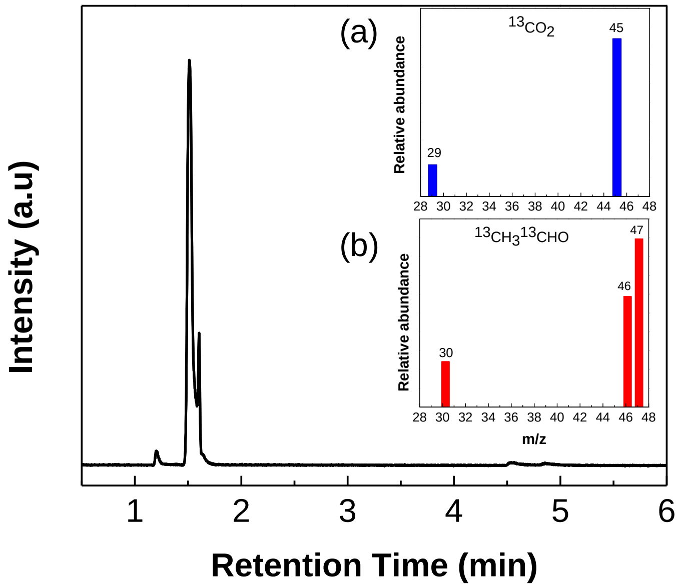

Supplementary Figure 12: Isotope tracer analysis. MS chromatograms and spectra of acetaldehyde produced by photocatalytic reduction of $^{13}CO_2$ with SnS_2-C. The insets show the mass spectra of (a) $^{13}CO_2$ gas and (b) acetaldehyde generated under $^{13}CO_2$ atmosphere.

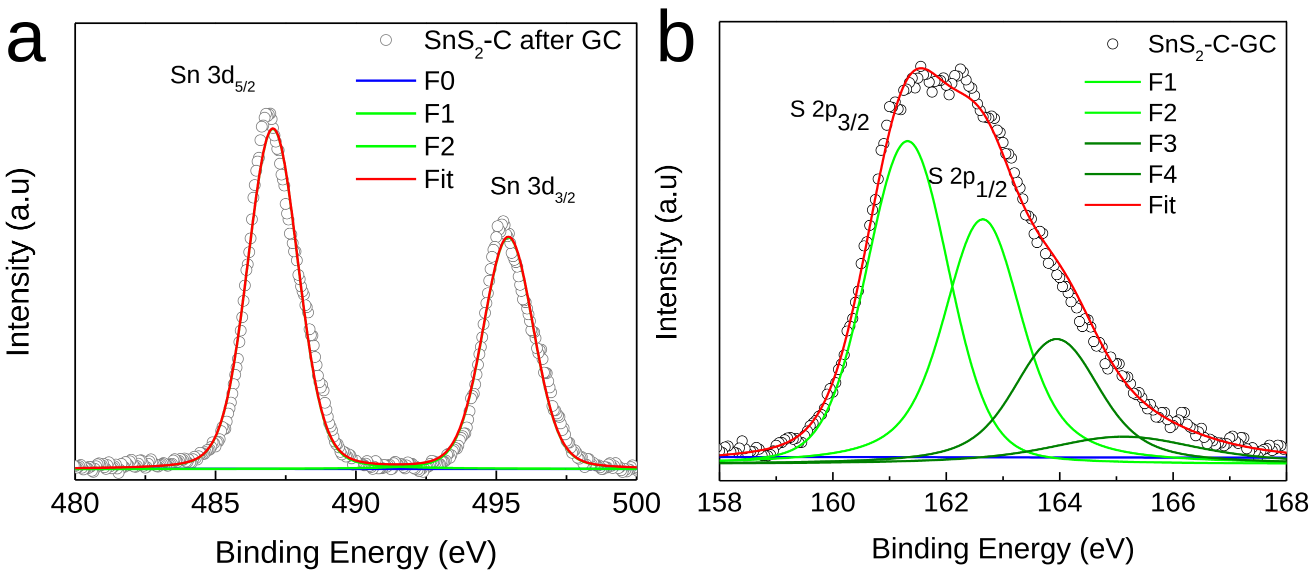

Supplementary Figure 13: Chemical composition analysis after photocatalytic reduction. a,b, High-resolution XPS spectra of SnS_2-C after photocatalytic performance for Sn 3d and S 2p, respectively.

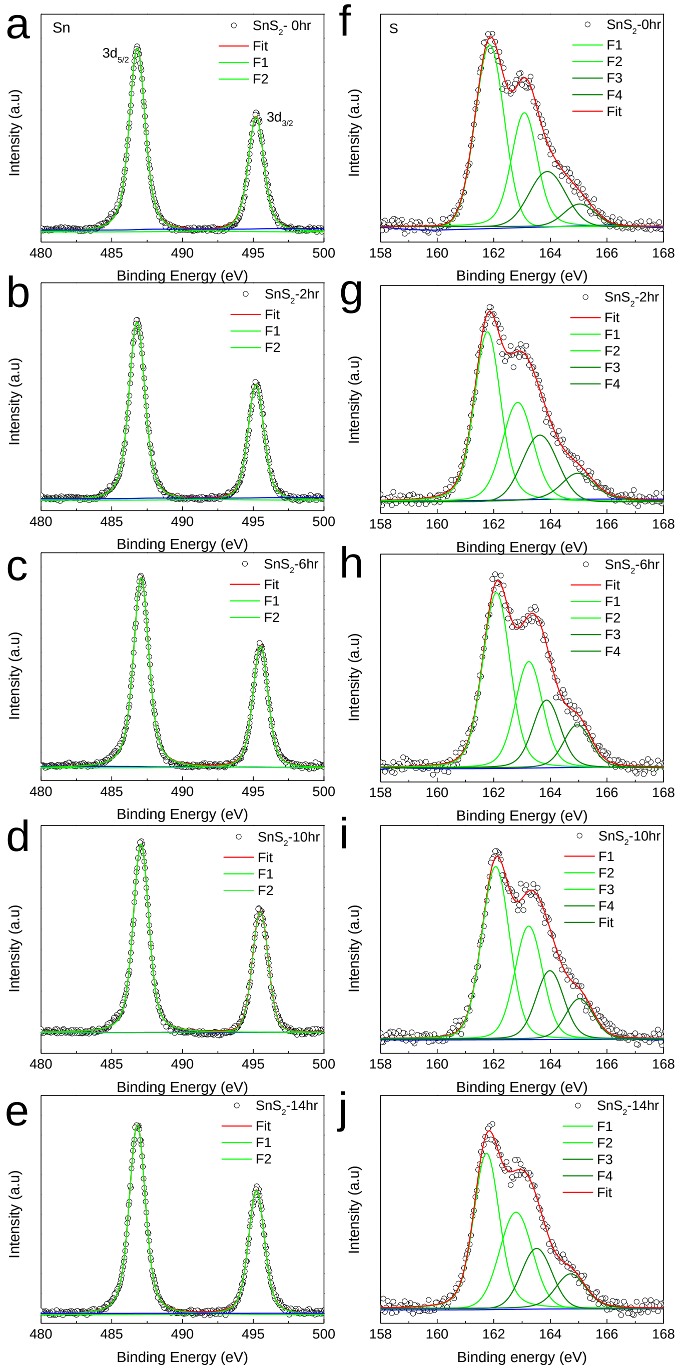

Supplementary Figure 14: Photocatalytic stability study by XPS analysis. High-resolution XPS spectra (a-e) Sn 3d and (f-j) S 2p with deconvoluted peaks of SnS_2-C before and after 2, 6, 10 and 14 hr. photocatalytic performance respectively.

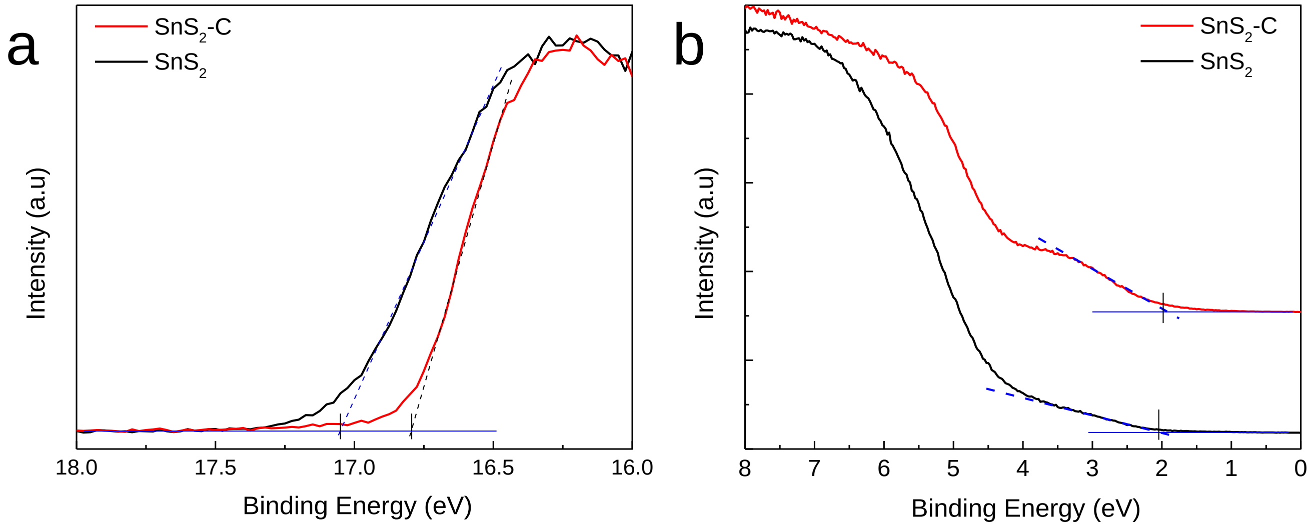

Supplementary Figure 15: Ultraviolet photoemission spectroscopy (UPS) study. Comparison of SnS_2-C and SnS_2 phases by photoemission spectroscopy. a,b UPS spectra taken with a photon energy of 21.2 eV: full valence band, with 0 eV binding energy corresponding to the Fermi level. Zoomed in views of the higher binding energy region (a) and low binding energy region (b).

Supplementary Table 1: Microstructural parameters of the SnS_2-C and SnS_2

**Sample**	**Peak**	**2θ**	**(D)= 0.9λ/βcosθ (nm)**	**Micro Strain ε (x 10^{-3}) = βcosθ/4 (*)**	**d=λ/2sinθ**
SnS_2	001	15.12	40.72	0.85	0.585
SnS_2	100	28.34	58.75	0.59	0.314
SnS_2	101	32.24	30.74	1.12	0.277
SnS_2	110	50.08	35.19	0.99	0.182
SnS_2-C	001	14.66	11.04	3.14	0.604
SnS_2-C	100	27.97	30.09	1.15	0.319
SnS_2-C	101	31.86	9.27	3.73	0.280
SnS_2-C	110	49.74	18.70	1.85	0.183

Supplementary Table 2: ^{13}C NMR chemical shift of L-cysteine and pyruvic acid

**^{13}C NMR chemical shift[1,2]**		
**Carbon**	L-cysteine	Pyruvic acid
**C1**	~27.5	~26.5
**C2**	~58.5	~162.1
**C3**	~174.8	~190.0

Supplementary Table 3: Formation energy and lattice constant calculation in carbon doped SnS_2 surface at interstitial/substitutional site by DFT-D2 method

Sample	Lattice constant (Å)			Formation Energy	Bader Charge (e)		
	a	b	c	E_f (eV)	Sn	S	C
**SnS_2**	11.049	11.049	26.775				
**C_{s-s}-1**	11.046	11.046	26.775	5.17	1.52	-0.77	-0.79
**C_{s-s}-2**	11.050	11.049	26.814	5.21	1.52	-0.77	-0.78
**C_{s-s}-3**	11.049	11.050	26.754	5.21	1.52	-0.77	-0.77
**C_{int}-1**	11.120	11.121	27.092	4.0	1.04	-0.24	-0.46
**C_{int}-2**	11.038	11.041	27.082	3.14	1.44	-0.30	-0.50
**C_{int}-3**	11.118	11.118	27.648	4.48	1.28	-0.26	-0.43
**C_{int}-4**	11.040	11.039	27.362	3.23	1.43	-0.36	-0.46
**C_{int}-5**	11.143	11.142	26.966	4.19	1.41	-0.75	-0.97
**C_{int}-6**	11.120	11.170	25.950	3.70	1.41	-0.34	-0.39
**C_{int}-7**	11.209	11.252	26.153	4.0	1.46	-0.40	-0.50

Supplementary Table 4: Photochemical Quantum Efficiency (PCQE) calculation

The GC measurement results with an area for the acetaldehyde peak, which is converted using standard acetaldehyde calibration.<br>$$\mathbf{PCQE} = \frac{\textbf{Mole of electron to convert hydrocarbons (HC) fuels}}{\textbf{Photons absorbed by catalyst}}$$<br>$$= \frac{\textbf{Number of electrons x Number of HC molecuels}}{\textbf{Number of photons absorbed by catalyst x Area}}$$<br>The PCQE (%) was determined by comparison of the maximum observed production rate of acetaldehyde with the measured photon flux. In total, 10 electrons are involved for the formation of acetaldehyde after reduction of CO_2.<br>$CO_2 + 10H^+ + 10e^- \rightarrow CH_3CHO + H_2O$ ……… $E^0 = -0.36V$	
**Light Source**	Halogen Lamp (300W)
**Photon Flux ($cm^{-2}.sec^{-1}$)**	4.54×10^{17}
**Product**	Acetaldehyde
**Catalyst loading**	100 mg
**Production rate (μmole/100mg.hr.)**	13.9
**Production rate (μmole/hr.)**	13.9
**Production rate (mole/hr.) (maximum)**	0.0000139
**Production rate (mole/sec)**	3.86×10^{-9}
**Number of molecule/sec**	$3.86 \times 10^{-9} \, [\frac{mole}{sec}] \, . \, 6.022 \times 10^{23} = 2.33 \times 10^{15}$
**No of electron for acetaldehyde**	10
**Area (cm^2)**	7.07
**PCQE (%)**	$\frac{100 \, x \, 10 \, x \, 2.33 \, x 10^{15}}{4.54 \, x \, 10^{17} x \, 7.07} = 0.72$

Supplementary Table 5: Wavelength dependent PCQE for SnS_2-C

**Band Pass Filter (nm)**	**PCQE (%)**
400 ± 25	1.64
500 ± 25	1.04
600 ± 25	0.32

Supplementary Table 6: XPS spectra analysis (relative areal peak intensity) before and after photocatalytic reaction

Sn (3d)					
**Peaks**	**0 hr**	**2 hr**	**6hr**	**10hr**	**14 hr**
**F1 ($3d_{5/2}$)**	61.50 %	61.38 %	62.74%	62.92	60.82 %
**F2 ($3d_{3/2}$)**	38.50 %	38.62 %	37.26%	37.07	39.18 %
**S (2p)**					
**Peaks**	**0 hr**	**2 hr**	**6hr**	**10hr**	**14 hr**
**F1**	46.61 %	42.31 %	44.67 %	43.79 %	42.31 %
**F2**	29.16 %	28.10 %	27.10 %	28.66 %	29.87 %
**F3**	17.73 %	19.56 %	17.27 %	17.33 %	17.03 %
**F4**	06.50 %	10.03 %	10.96 %	10.21 %	11.08 %

Supplementary Table 7: Work function and VBM of the SnS_2-C and SnS_2 (vs vacuum level)

	**SnS_2**	**SnS_2 -C**
**Onset**	17.04 eV	16.8 eV
**Work function**	4.16 eV	4.4 eV
**VBM**	2.05 eV	1.99 eV

Supplementary Methods

Density functional theory (DFT) computational methods. In this study, we considered two possible ways of introducing carbon doping atom into the 2D SnS_2 2H polytype and CO_2 adsorption and reduction on carbon doped SnS_2. All present calculations were performed with the DFT plane-wave method utilizing the Vienna ab initio simulation package (VASP)[3]. The generalized gradient approximation (GGA) parameterized by the Perdew-Burke-Ernzerhof (PBE) along with projector augmented wave method (PAW) was used for total energy calculation[4,5]. The valence electron configurations considered in this calculation are Sn($4d^{10}5s^25p^2$), S($3s^23p^4$), C($2s^22p^3$) and O($2s^22p^4$) respectively. To account for the *van der Waals* interactions between the SnS_2 layers, we have utilized the standard PBE functional augmented by the Grimme's semi-empirical dispersion potential corrected DFT-D2 method[6]. The convergence criterion for the self-consistent iteration was 1 x 10^{-6} eV. The calculations were carried out with a 500 eV cutoff energy for the plane wave basis set. The k-point grid for the integration in the Brillouin zone of the hexagonal lattice using the (8x8x5) and (5x5x2) Gamma centered for bulk and surface, respectively, was generated for the structural optimization and calculation of the electronic properties. 18 [SnS_2] units doped with one C atom were modeled by (3×3) supercell slabs separated perpendicularly by a 20.0 Å vacuum space. All positions and lattice constants were fully relaxed and optimized up to a force convergence of 0.01 eV $Å^{-1}$. Atomic charges of the optimized structures were calculated by utilizing the Bader method with a program designed by Henkelman et al.[7].

Model structure and carbon doping. The experimental hexagonal 2H polytype of SnS_2 has a layered crystal structure with a space group symmetry (number 164) of $P\bar{3}m1$ with the lattice constants a = 3.649 Å and c= 5.899 Å[8-10]. To test the reliability of our calculations, the methods and parameters employed in the current work were first examined by optimizing the bulk SnS_2 using the PBE method and the lattice parameters, a = 3.701 Å, c = 6.590 Å, are larger than the experimental data. The SnS_2 unit cell is clearly linked to the van der Waals interactions (vdW)

between the S–Sn–S sandwich layers with an Sn atom coordinating to six S atoms in an octahedral environment. They are important for describing the interaction between SnS_2 layers. Our results for lattice constant is a = 3.689 Å, c = 5.882 Å using the DFT-D2 functional, in good agreement with the experimental values[8-10]. The following discussions will be based on the results of the calculations with the DFT-D2 method.

To model the carbon doped material, we considered a SnS_2 supercell model containing 54 atoms and the cell volume of 11.049 Å x 11.049 Å x 26.775 Å as shown in Supplementary Figure 8. As aforementioned, we considered two possible ways of introducing C-doping atom into the lattices of SnS_2. The first was an S atom substituted with a carbon atom (hereafter denoted by $C_{S\text{-}S}$); the second was a C doping atom sited in an interstitial position (hereafter denoted as C_{int}). The atomic ratio of the doping concentration was calculated by using C to Sn. For example, in one doping case 1 C atom to 18 Sn atoms, corresponding to 5.5%. To characterize the stability of the doped SnS_2, we calculated the formation energies (E_f) of a dopant atom in substitutional and interstitial configurations using the following relation

$$\mathbf{E_f = E_{(doped\text{-}SnS2)} - (E_{SnS2} - \delta_{\mu x} + \mu_C) - 1} \ldots\ldots\ldots\ldots\ldots\ldots(1)$$

Where E_{SnS2} and $E_{doped\text{-}SnS2}$ are the total energies of pristine SnS_2 supercell and doped SnS_2. The quantities μ_C and μx represent the chemical potentials of carbon and substituted atom in the host lattice (x = Sn or S), respectively. The chemical potential for doping carbon, μ_C, is calculated from the graphene sheet as the total energy of bulk carbon sheet/number of carbon atoms. The chemical potential for host atoms S and Sn atom energies were calculated with respect to the alpha solid S8[11] and bulk Sn metal. In the formation energy calculations, in Eq.1, the interstitial doping case, the term μx was omitted (δ=0); while in the substitutional doping δ=1. The formation energies of dopant atom in different configurations were calculated by using DFT-D2 method and presented in Supplementary Table 4. The supercell model and partial geometries from the structurally optimized C-doped SnS_2 are shown in Supplementary Figure 8. We first determined the carbon atom substituted with an S atom in a top layer of SnS_2 supercell; this partial geometry is shown in Supplementary Figure 8.

We start by analyzing the substitution of S by C in three different sulphur layers in the SnS_2 supercell; the first model represents one S atom replacing by one C atom, forming three C-Sn bonds with surrounding Sn ions at the top and bottom of the first layer, which are denoted as C_{S-S}-1 and C_{S-S}-2, respectively. The other substitution at the top of the second layer is C_{S-S}-3. The calculated formation energies for the respective C substitutions are ranging from 5.17 to 5.21 eV. The formation energies of the dopant atom in different configurations are presented in Table S3. This substitution leads to significantly shorter Sn-C bonds (2.234 Å), with respect to the original Sn-S bond (2.590 Å). After the C substitution at the top of the first layer, the optimized lattice constant of the C_{S-S}-1 is a=11.046 Å and c=26.775 Å, almost similar to that of the SnS_2 supercell (a=11.046 Å and c=26.775 Å). Using the Bader charge analysis method, we computed the charges of the doping C atom and neighboring Sn and S atoms in the 2D SnS_2 supercell as shown in Supplementary Table 3, which shows that the charge on the C ion in the substitutional C atom to S in C_{S-S}-1 is -0.79 e, and the adjacent Sn and S atom charges are 1.52 e and -0.77e.

In the interstitial model (C_{int}), we have investigated the effect of C doping located interstitially at various places in between the S and Sn layers of SnS_2 supercell and their geometries are shown in Supplementary Figure 8. We first calculated the C atom doping interstitially located in between S and Sn top layers, and the results show that the C atom is bound to three S atoms in the SnS_2 with the bond lengths C-S and C-Sn predicted to be 1.723 Å and 3.062 Å, respectively, and their formation energy is 4.0 eV. Among the all interstitial doping calculations, the C atom bounds to three lattice S atoms at the top layer forming one C-Sn bond, which is C_{int}-2 and has the lowest formation energy compared to others configurations. From these results, it can be clearly seen that the lattice parameters of all the interstitial C doped SnS_2 models are found to increase only the c-axis of SnS_2 (slightly increased in a and b axes). Our calculated energies indicate that the interstitial C doping has the lowest formation energy than the substituted ones as shown in Supplementary Table 3.

Photochemical CO_2 reduction experiment. The photocatalytic experiment for the reduction of CO_2 was performed at ambient temperature (25 ± 5 ºC) in a continuous gas flow reactor. The volume of the cylindrical reactor which was made of stainless steel and covered with Quartz-Glass was 300 ml (11 cm x 4 cm). One sample dish containing 0.1 g of the photocatalysts obtained by the method of the present study was placed in the middle of the reactor. A 300 W commercial halogen lamp was used as the light source. The lamp was vertically placed outside the reactor above the sample dish. Two mini fans were fixed around the lamp to avoid the temperature rise of the flow system. The reactor was firstly degassed at 50 ºC and then purged with nitrogen (N_2) (30 sccm) for 1 hour in order to remove any residual compounds inside the reactor surface. The catalyst powder spread onto the glass disc, with a diameter of around 4 cm. Initially nitrogen gas was purged (4 sccm) inside the reactor for 1 hour to remove the air with other gases. After that, CO_2 was purged inside the reactor for another 1 hour and flow rate was controlled at 4 sccm. The CO_2 was flowing through water to control the desired humidity level for the entire experiment. The halogen lamp was turned on after one hour while adsorptions and desorption of gas and photocatalyst reached the equilibrium. The concentration of hydrocarbon products was continuously measured by a GC-FID in vapor phase. All photocatalytic tests were performed over a period of 13 hours of irradiation. The detail schematic drawing of the experimental setup is shown in Supplementary Figure 10.

Isotope tracer analysis. The photocatalytic isotope tracer analysis experiment for the reduction of CO_2 was performed at ambient temperature (25 ± 5 ºC) in a batch type close reactor. The volume of the cylindrical reactor which was made of stainless steel and covered with Quartz-Glass was 300 ml (11 cm x 4 cm). One sample dish containing 0.1 g of the photocatalysts obtained by the method of the present study was placed in the middle of the reactor. A 300 W commercial halogen lamp was used as the light source. The lamp was vertically placed outside the reactor above the sample dish. Two mini fans were fixed around the lamp to avoid the temperature rise of the flow system. The reactor was firstly degassed at 50 ºC and then purged with nitrogen (N_2) (30 sccm) for 3 hour in order to remove any residual compounds inside the reactor surface. The catalyst powder was spread onto the glass disc, with a diameter of around 4 cm. Initially nitrogen gas was purged (4 sccm) inside the reactor for 1 hour to remove the air with other gases. After that, $^{13}CO_2$ was purged inside the reactor for another 20 min; after that we

closed the reactor outlet and continued the $^{13}CO_2$ gas flow for another 30 min. The CO_2 was flowed through water with a desired humidity level for the entire experiment. The halogen lamp was turned on after one hour while adsorptions and desorption of gas and photocatalyst reached the equilibrium. The gas chromatography-mass spectrometry (GC-MS) analysis of the hydrocarbon products was performed by a GC (HP6890)/MS(5973) system by manually collected reaction gas mixture from batch type reactor after 3 hours of irradiation.

Ultraviolet photoemission spectroscopy (UPS) study. Supplementary Figure 15 shows the valence band spectra of the SnS_2-C and SnS_2. The zoomed in views of the higher binding energy region (a) and low binding energy region (b) correspond to the onset and valence band maximum (VBM) of the SnS_2-C and SnS_2, respectively. The calculated work function and VBM of SnS_2-C and SnS_2 are summarized in Supplementary Table 7.

Supplementary References:

1 Abraham A., Mihaliuk E., Kumar B., Legleiter J., and Gullion T. Solid-State NMR Study of Cysteine on Gold Nanoparticles. *J. Phys. Chem. C*, **114**, 18109-18114, (2010).

2 Meyer W., Heckmann J., Hess C., Radtke E., Reicherz G., Triebwasser L., Wang L. Dynamic polarization of 13C nuclei in solid 13C labeled pyruvic acid. *Nuclear Instruments and Methods in Physics Research A*, **631**, 1-5 (2011)

3 Kresse, G. & Furthmüller, J. Efficient iterative schemes for \textit{ab initio} total-energy calculations using a plane-wave basis set. *Physical Review B* **54**, 11169-11186 (1996).

4 Blöchl, P. E. Projector augmented-wave method. *Physical Review B* **50**, 17953-17979 (1994).

5 Perdew, J. P., Burke, K. & Ernzerhof, M. Generalized Gradient Approximation Made Simple. *Physical Review Letters* **77**, 3865-3868 (1996).

6 Grimme, S. Semiempirical GGA-type density functional constructed with a long-range dispersion correction. *Journal of Computational Chemistry* **27**, 1787-1799, (2006).

7 Henkelman, G., Arnaldsson, A. & Jónsson, H. A fast and robust algorithm for Bader decomposition of charge density. *Computational Materials Science* **36**, 354-360,(2006).

8 Podberezskaya, N. V., Magarill, S. A., Pervukhina, N. V. & Borisov, S. V. Crystal chemistry of dichalcogenides MX2. *Journal of Structural Chemistry* **42**, 654-681, (2001).

9 Burton, L. A. *et al.* Electronic and optical properties of single crystal SnS_2: an earth-abundant disulfide photocatalyst. *Journal of Materials Chemistry A* **4**, 1312-1318,(2016).

10 Filso, M. O., Eikeland, E., Zhang, J., Madsen, S. R. & Iversen, B. B. Atomic and electronic structure transformations in SnS_2 at high pressures: a joint single crystal X-ray diffraction and DFT study. *Dalton Transactions* **45**, 3798-3805,(2016).

11 Rettig, S. J. & Trotter, J. Refinement of the structure of orthorhombic sulfur, [alpha]-S8. *Acta Crystallographica Section C* **43**, 2260-2262, (1987).
